# High-Selective Extraction of Scandium (Sc) from Bauxite Residue (Red Mud) by Acid Leaching with MgSO_4_

**DOI:** 10.3390/ma15041343

**Published:** 2022-02-11

**Authors:** Andrei Shoppert, Irina Loginova, Julia Napol’skikh, Dmitry Valeev

**Affiliations:** 1 Laboratory of Advanced Technologies in Non-Ferrous and Ferrous Metals Raw Materials Processing, Ural Federal University, 620002 Yekaterinburg, Russia; 2 Department of Non-Ferrous Metals Metallurgy, Ural Federal University, 620002 Yekaterinburg, Russia; i.v.loginova@urfu.ru (I.L.); anapolskikh512@gmail.com (J.N.); 3 Laboratory of Sorption Methods, Vernadsky Institute of Geochemistry and Analytical Chemistry of the Russian Academy of Sciences, 119991 Moscow, Russia; dmvaleev@yandex.ru

**Keywords:** red mud, waste utilization, rare earth elements, magnesium sulphate, acid leaching, selectivity, kinetics

## Abstract

Bauxite residue, also known as red mud (RM), from alumina production is the most promising technogenic material for the production of scandium (Sc) and other rare earth elements (REEs). Conveniently, RM is processed by using a strong acid (pH < 2.5), which lead to co-dissolution of iron and other undesirable major components. In this work, for the first time, the possibility of selective extraction of scandium from red mud by using highly diluted acid (pH > 4) in the presence of MgSO_4_ was shown. The effect of temperature (40–80 °C), time (0–60 min), pH (2–5), and the MgSO_4_ concentration (12–36 g L^−1^) on Sc extraction efficiency was evaluated. It was shown that Sc extraction was higher than 63% even at a pH of 4, at 80 °C, after 1 h, while more than 80% could be extracted at a pH of 2. Iron extraction reduced from 7.7 to 0.03% by increasing the pH from 2 to 4. The kinetics study using the shrinking core model (SCM) has shown that diffusion through a product layer is a rate-limiting stage of the process at high temperatures (>60 °C) and low pH (<3), whereas, at lower temperatures and higher pH values, the leaching rate is limited by diffusion through the liquid film.

## 1. Introduction

Red mud (RM) is a waste generated during bauxite processing for alumina production. The most common method for bauxite processing is the Bayer process, where raw material is digested under high pressure by NaOH to obtain a pregnant sodium aluminate solution [1]. During high-pressure leaching, non-soluble elements such as iron, titanium, and refractory or precipitated during washing step aluminum minerals, as well as sodium aluminum hydrosilicates (Na_6_Ca_2_(AlSiO_4_)_6_(CO_3_)_2_) and hydrogarnets (Ca_3_Al_2_Si_3_(OH)_12_) formed by the reaction of lime and silica, remain in the form of solid residue. Then, this solid residue is stockpiled on the red mud landfills that cause severe harm to the environment [2]. The amount of RM accumulated worldwide already exceeds 150 billion tons, and every year this amount increases by another 150 million tons [3]. At the same time, RM is an interesting source of rare earth elements (REEs) [4,5,6,7,8,9], especially scandium (Sc) which represents more than 90% of the REE value in this solid residue [10]. The Sc concentration in typical RM is about 90–120 mg kg^−1^ [11,12], which is five times higher than the content in the Earth’s crust [13]. However, Sc concentration can reach more than 200 mg kg^−1^ in some types of RM with a very low yield (lower than 30% of the raw bauxite mass) [14].

The acidic methods are commonly proposed for extracting minor elements from RM [10,15,16,17,18,19,20]. These methods allow a high recovery of REEs by leaching with inorganic and organic acids. Previous research has shown [13] that, by using a strong acid (pH < 2.5), the extraction of REEs is more than 50%. However, the co-dissolution of Fe occurs because some of the REEs are entrapped in the solid matrix of iron minerals [10]. Therefore, a large acid consumption is needed. Co-dissolution of a significant amount of the major components (Fe, Ca, Al, and Si) also makes it difficult to recover Sc and other REEs from the leaching solution [21]. Therefore, recent studies have focused on selective REE extraction from RM [11,22,23], which is key for cost-effective technologies development.

To extract REEs from the solid matrix of iron minerals, the use of sintering methods has been proposed [24]. During the sintering of RM with acid or sodium species, iron minerals are transformed into a new phase with the release of REEs. Subsequent REE extraction could be performed more easily and using mild conditions, i.e., acid leaching at a pH value higher than 2.5 [25]. Our previous work showed [14] that Sc extraction from the sintering RM is closely connected with magnesium extraction. In recent works by Xiao Y. et al. [26,27], it was shown that Mg^2+^ ions could serve as a leaching agent to extract REEs from ion-adsorption type rare earth ore. Therefore, Sc and other REEs in the sinter RM were most likely adsorbed on the solid residue surface after their release from the solid matrix.

In this paper, we aim to evaluate the MgSO_4_ leaching of Sc and other REEs from the RM obtained by water-alkali leaching of the sintering process dust that was performed in our previous research [28]. This RM contains up to 240 mg kg^−1^ Sc and a high amount of other REEs (up to 3200 mg kg^−1^) that have been released from the iron minerals solid matrix during the sintering process. The effects of temperature, time, pH, and MgSO_4_ concentration on Sc extraction were studied. The kinetics of the leaching process was also evaluated using the shrinking core model (SCM). The solid residue was characterized using SEM-EDX analysis. The data obtained in this research should help to better understand the mechanism and kinetics of effective and high-selective leaching of REEs from RM using a leaching agent which was novel to this raw material.

## 2. Materials and Methods

### 2.1. Materials and Reagents

The red mud (RM-ESPD) used in this research was obtained by water-alkali leaching of the electrostatic precipitator dust (ESP dust) formed during the bauxite sintering process. The ESP dust was obtained from the RUSAL-Kamensk-Uralsky alumina refinery (GPS coordinates: 56.304530, 61.980334, Kamensk-Uralsky, Russia), where low-grade bauxite is processed via a combined Bayer-Sintering process. Bauxite with high silica content is sintered in a rotary kiln together with sodium carbonate (Na_2_CO_3_) and lime (Ca(OH)_2_) to obtain water-soluble sinter at 1150 °C. During the process, especially at the drying and decarbonization stages, a high number of fine particles were formed that left the kiln together with exhaust gases. An electrostatic precipitator captured the tiniest particles (less than 20 μm) from the exhaust gases. The ESP dust chemical and phase compositions were shown in a previous research [28]. The ESP dust water leaching was carried out by distilled water at a temperature of 95 °C, L/S ratio of 10:1, and leaching time of 60 min. The solid residue from water leaching was subjected to high-pressure alkali leaching by sodium aluminate solution (Na_2_O (caustic alkali) = 240 g L^−1^, Al_2_O_3_ = 120 g L^−1^) at a temperature of 200 °C, L/S ratio of 3:1, and leaching time of 90 min. Sodium aluminate solution was obtained by dissolving predetermined amount of NaOH and Al(OH)_3_ in distilled water, then the solution was filtered and diluted to desired concentration. After alkali leaching, the resulting RM-ESPD was separated from the aluminate solution by filtration, followed by distilled water washing. After washing, the RM-ESPD was dried for 8 h at 110 °C, and used for analysis.

All reagents (H_2_SO_4_, MgSO_4_, NaOH, and Al(OH)_3_) used in this study were of analytical grade. Distilled water was obtained using a GFL distiller (GFL mbH, Burgwedel, Germany).

### 2.2. Analysis

The mineral compositions of the raw red mud and solid residue were determined by X-ray diffraction (XRD) on a Rigaku D/MAX-2200 diffractometer (Rikagu Corp., Tokyo, Japan) using the “Match! 3” software (Version 3.12, Crystal impact, Bonn, Germany). The semi-quantitative analysis of crystalline phases in the RM-ESPD was carried by the Rietveld method using “Match! 3” software. The compositions of major compounds in the RM-ESPD and the solid residue were analyzed via the X-ray fluorescence (XRF) method using an Axios MAX X-ray fluorescence spectrometer (Malvern Panalytical Ltd., Almelo, The Netherlands). The concentration of REEs, in the raw red mud and leachate, was determined using inductively coupled plasma mass spectrometry (ICP-MS) on a PerkinElmer NexION 300S instrument (PerkinElmer Inc., Waltham, MA, USA). The ICP-MS analysis of the solid samples was performed after complete dissolution by a mixture of concentrated hydrofluoric (HF), sulfuric (H_2_SO_4_), and nitric (HNO_3_) acids (SigmaTek, Moscow, Russia).

The morphology of the solid samples and the elemental composition of the main minerals were determined using scanning electron microscopy with energy-dispersive X-ray spectroscopy (SEM-EDS, Vega III, Tescan, Brno, Czech Republic). The particle size distribution of the RM-ESPD and the solid residue was determined by the laser diffraction method (LD) using a SALD-7500 (Shimadzu, Kyoto, Japan).

### 2.3. Experimental

The leaching of the RM-ESPD by MgSO_4_ acidified solutions with concentrations of 12, 24, and 36 g L^−1^ was carried out at a L/S ratio 10 L kg^−1^; at temperatures of 40, 50, 60, 70, and 80 °C; at pH values of 2, 3, 4, and 5; for 60 min; and in a 0.5 L Lenz Minni thermostatic reactor (Lenz Laborglas GmbH & Co. KG, Wertheim, Germany) fitted with a three-necked lid for overhead stirrer, a condenser, and sampler. The pH of the solution was kept constant by the addition of 1 M H_2_SO_4_ using automatic titrator ATP-02 (Akvilon, Podolsk, Russia). During the leaching, a portion of the pulp was taken every 5 min within the first 0–20 min period and every 10 min within the second 20–60 min period. The pulp was filtered using a syringe filter (pore size of 0.45 um) and diluted with 3% HNO_3_ before the ICP-MS analysis for the concentration of REEs in the leachate.

After 60 min of leaching, the solid residue was separated from the solution by filtration on a Buchner funnel. After washing and drying of the solid residue for 8 h at 110 °C, the contents of major components, morphology, and particle size distributions were evaluated using XRF, SEM-EDX, and LD methods. The extraction of REEs was calculated by using the following equation:

ε_REE_ = [(C_REE_ × V_s_)/[REE] × m_RM_] × 100%, (1)

where ε_REE_ is the degree of REE extraction, %; C_REE_ is the REE content in solution after leaching, mg L^−1^; V_s_ is the volume of solution, L; [REE] is the REE content in raw RM, mg kg^−1^; and m_RM_ is the weight of raw RM, kg.

Fitting the experimental data to the model equations was carried out using the graphical least squares method using the "nonlinear curve fit analysis" function in the commercial software.

## 3. Results and Discussion

### 3.1. Red Mud Characterization

As was shown in our previous research [28], water-alkali leaching of ESP dust leads to the formation of RM with high REE concentrations. To reveal the phase composition and the composition of selected elements of the RM-ESPD, X-ray diffraction, X-ray fluorescence, and scanning electron microscopy analysis were used.

**Figure 1:**
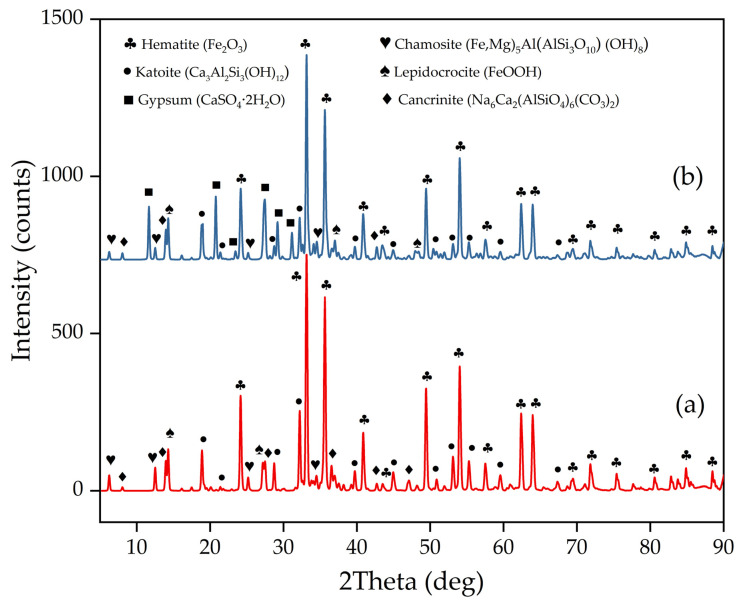
XRD patterns of: (**a**) The red mud obtained by water-alkali leaching of electrostatic precipitator dust arising from the bauxite sintering process (RM-ESPD); (**b**) solid residue after MgSO_4_ leaching.

Figure 1a shows the XRD pattern of the raw RM-ESPD. As can be seen, the raw red mud consists of hematite (Fe_2_O_3_), katoite (Ca_3_Al_2_Si_3_(OH)_12_), chamosite ((Fe,Mg)_5_Al(AlSi_3_O_10_)(OH)_8_), lepidocrocite (FeO(OH)), and a low amount of cancrinite (Na_6_Ca_2_(AlSiO_4_)_6_(CO_3)2_). This mineral composition is close to that of the Bayer RM [29,30,31]. However, the amount of cancrinite is higher in the industrial RM and there is no lepidocrocite. A low amount of cancrinite can be attributed to the fact that Na_2_SiO_3_ was dissolved from ESPD by water before Al extraction. Lepidocrocite was formed via the water leaching of the dust that contained sodium ferrite formed during the sintering of bauxite with soda (Na_2_CO_3_). However, ESPD passes very quickly through the hot zones of the furnace, and the reaction of hematite with soda fails to complete. Therefore, the main iron mineral in the RM-ESPD is hematite. It was confirmed by the semi-quantitative analysis performed via Rietveld methods (Table 1).

In addition to soda, lime is added in the bauxite sintering process to convert silica into insoluble calcium silicate. During high-pressure alkali leaching, lime reacts with the sodium aluminate and sodium silicate with the formation of hydrogarnet, katoite. Some parts of sodium aluminate and sodium silicate precipitate in the form of cancrinite (Figure 1 and Table 1). Thus, a high amount of Ca and Na could be seen in the RM-ESPD according to X-ray fluorescence analysis (Table 2).

Table 2 shows that iron (III) oxide is the major oxide in the RM-ESPD, followed by silica, calcium oxide, alumina, titanium oxide, and sodium oxide. Some amount of iron (II) oxide could be found in the chamosite. Non-metallic elements, especially carbon oxide, may also be present in this type of RM. The RM-ESPD contains a relatively high amount of REEs. The concentration of selected REEs in the RM-ESPD is shown in Table 3. It can be seen that the concentration of Sc (and other REEs) in this type of RM is very high: 240 mg kg^−1^ vs. 100 mg kg^−1^ for the RM obtained from the same bauxite by the Bayer process [32]. According to our previous research using electron probe microanalysis [28], Sc in the RM-ESPD is mainly associated with iron minerals. Borra et al. [10] showed that REEs in RM are adsorbed on iron minerals or entrapped in their solid matrix. The RM-ESPD was subjected to high temperature in the presence of soda and lime. Therefore, it could be suggested that REEs can be liberated from the solid matrix of iron minerals and represented in this RM in the ion-exchange phase that was adsorbed on iron minerals or in the tunnels of the desilication products [33,34].

**Figure 2:**
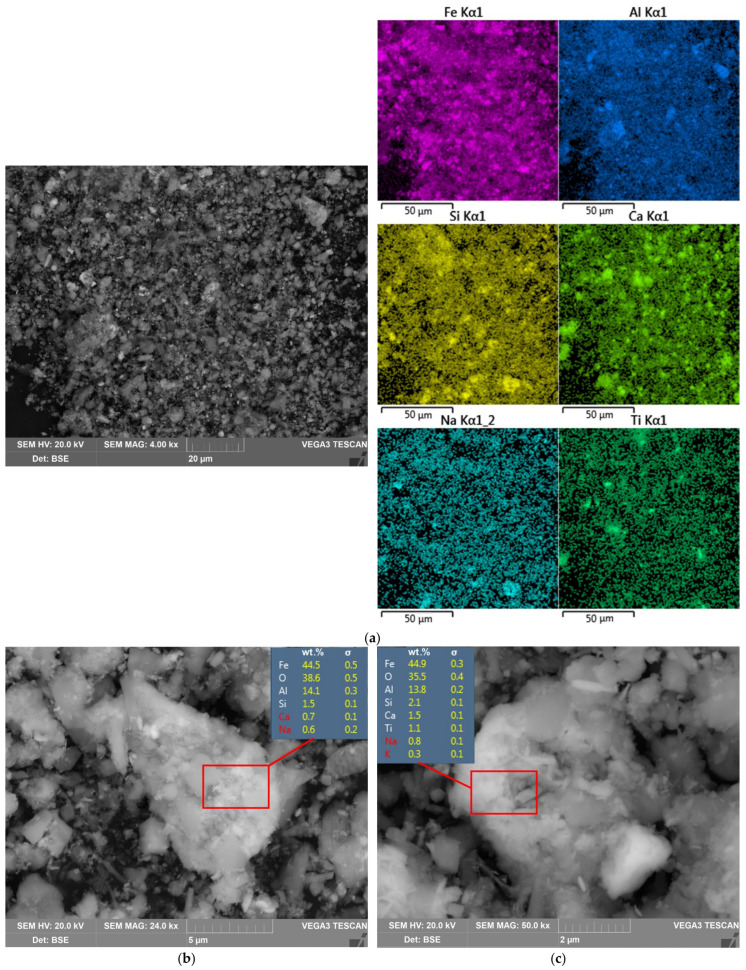
Back-scattered electron (BSE) image of: (**a**) the RM-ESPD surface and elemental distribution of major elements (EDS mapping); (**b**) particles of the RM-ESPD at the magnitude of 24,000×; (**c**) particles of the RM-ESPD at the magnitude of 50,000×.

**Figure 3:**
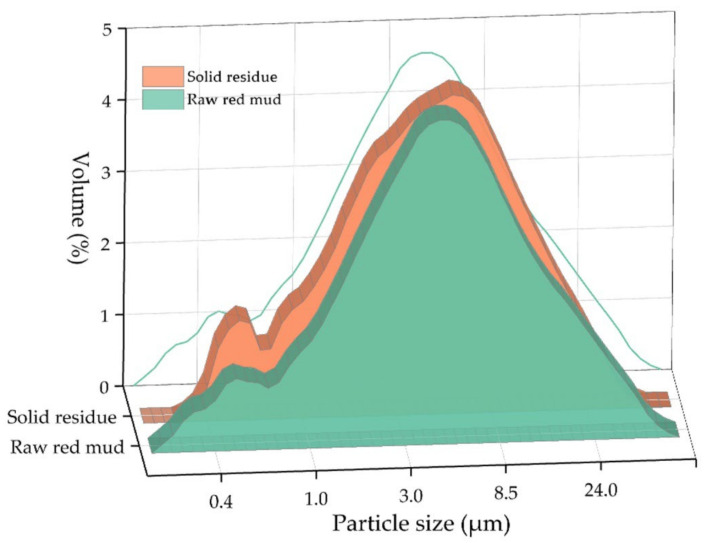
Particle size distribution (PSD) of the RM-ESPD and solid residue after MgSO_4_ leaching (green line is the projection of the raw red mud PSD on the ZY plane).

In Figure 2, the SEM-EDS images of the RM are shown. As can be seen, the RM-ESPD consists of very fine particles with different shapes. The RM-ESPD was obtained from the smallest fraction of the sintering process dust, i.e., electrostatic precipitator dust. Therefore, particle sizes vary from 1 to 10 μm. It is also confirmed by LD analysis, as shown in Figure 3. According to LD, 80% of the RM-ESPD particles are less than 10 μm, and only 10% of the particles are less than 1 μm. In comparison, the particle size of the Bayer RM generally ranges between 10 and 100 μm [29].

The high concentration of iron and the association of Ca and Na with aluminosilicates in the RM-ESPD was confirmed by SEM-EDS mapping (Figure 2a). However, grains that mainly consist of Ca oxides are depicted in Figure 2a. They can be represented by unreacted calcite. According to mineralogical composition (Figure 1 and Table 1), the most abandon iron mineral in this type of RM is hematite with a low amount of iron hydroxide. Figure 2b,c shows that hematite particles are covered by DSP (cancrinite and katoite—aluminum containing minerals).

Lin et al. [35] reported that the reduction in particle size and preliminary alkali and/or thermal activation greatly improved REE extraction from coal-related materials by mineral acids. As can be seen from the RM characterization, the RM-ESPD was already alkali and thermally activated; moreover, its particles were very fine. Therefore, high REE extraction efficiency is expected even under mild leaching conditions.

### 3.2. Leaching with MgSO_4_

**Figure 4:**
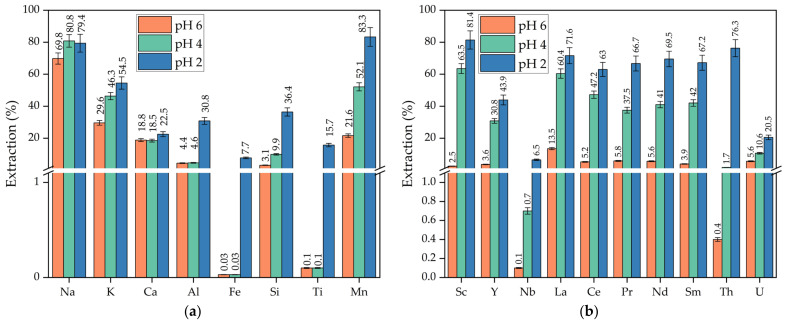
Effect of pH at a temperature of 80 °C, leaching time of 60 min, L/S ratio of 10:1, and MgSO_4_ concentration of 24 g L^−1^ on: (**a**) the extraction of major elements; (**b**) the extraction of minor elements.

Xiao, Y. et al. [26] showed that extraction of REEs from ion adsorption-type rare-earth ore by MgSO_4_ can be accomplished at a pH of 5.7. However, leaching of REEs from industrial RM is very low at pH values > 2.5. To study the effect of pH on the extraction of REEs from the RM-ESPD by MgSO_4_, leaching experiments were conducted at pH values of 2, 4, and 6; temperature of 80 °C; leaching time of 60 min; L/S ratio of 10:1; and MgSO_4_ concentration of 24 g L^−1^. The degree of extraction of major and minor elements from the RM-ESPD is shown in Figure 4. The concentrations of major and minor elements in the leaching solution are shown in Table 3. Figure 4a shows that the pH value greatly affects Fe, Ti, Si, and Al extraction. The extraction of Fe and Ti was lower than 0.1% at a pH of 4–6. The extraction of REEs using solution with a pH of 4 was lower than at a pH of 2 by 10–20%. As was shown in the literature [10,13,17] and in our previous research [14], Sc extraction by different acids was very low at pH values higher than 2.5 However, in this work, the extraction of almost all selected REEs was lower than 10% only at a pH of 6. Such low extractions of REEs, Fe, Al, and Ti using diluted acids are connected with the low accessibility of the REEs by H^+^ ions in the solid matrix of iron minerals that remain unleached at this pH, The possible reason for this is connected with the pH of their hydroxides precipitation, i.e., Fe(OH)_3_ begins to precipitate at a pH of 3 [35]. Sc(OH)_3_ begins to precipitate at a pH higher than 6. If Sc is represented in the raw material by ionic-type minerals, then it can be extracted by MgSO_4_ even at a pH higher than 3 [26]. Therefore, using MgSO_4_ and the pH range of 3–4, a solution can be obtained with the Fe and Ti concentrations lower than 20 mg L^−1^ (Table 4), with the extraction of more than 63.5 wt.% of Sc (Figure 4). The concentrations of Th and U were also very low at pH 4. It should be noted that these elements make it difficult to recover REEs from the solution [21]. The low extraction of Nb and Y could be attributed to the formation of insoluble compounds at high pH values [35].

**Figure 5:**
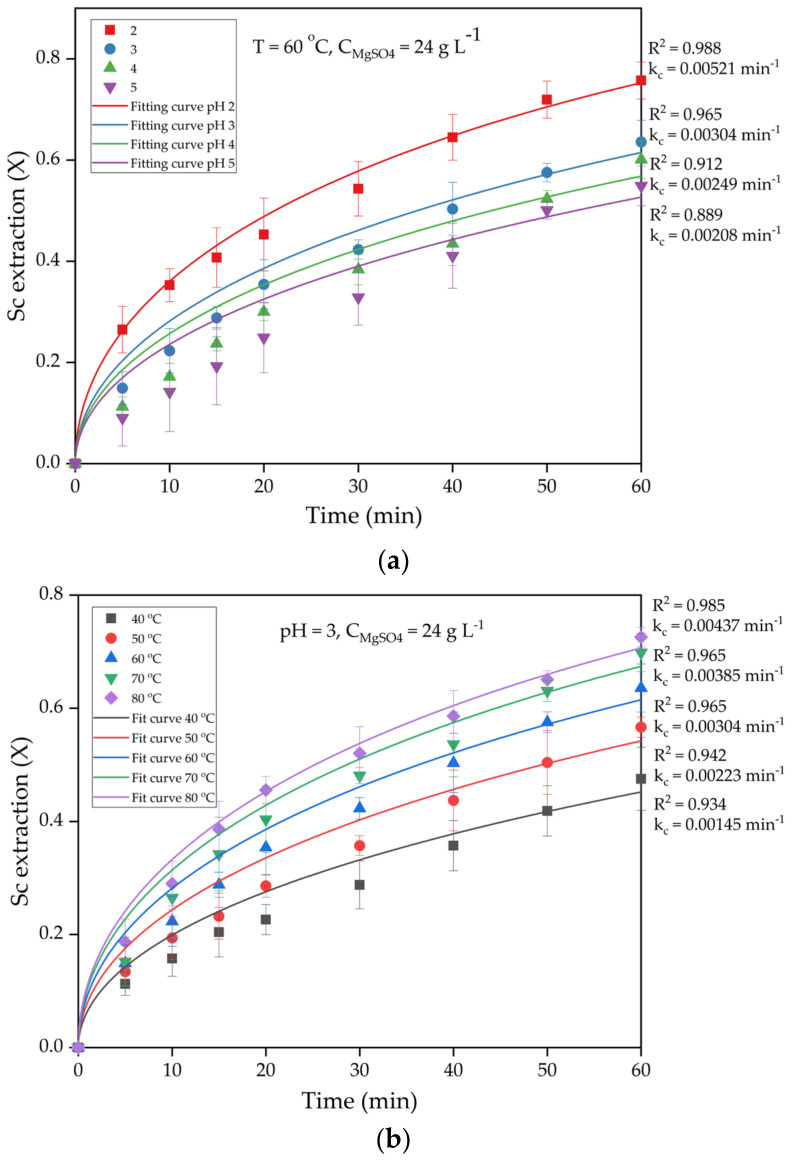

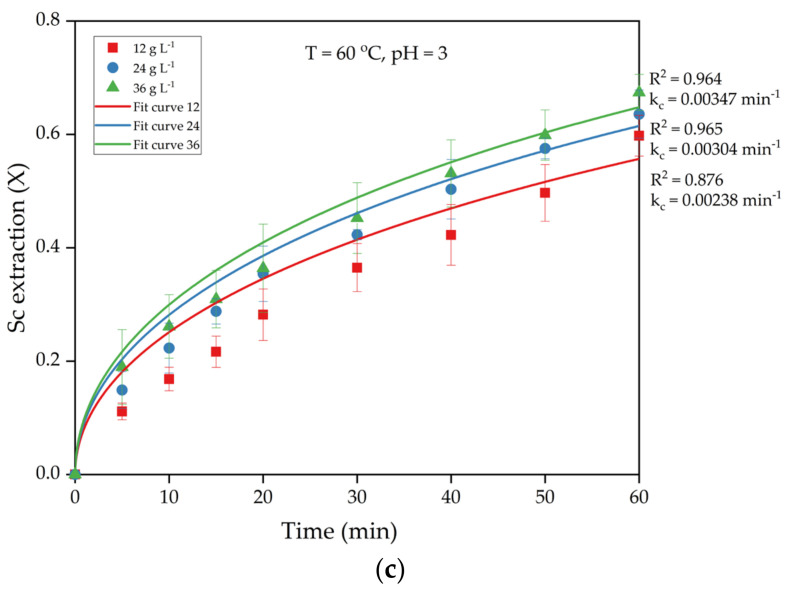
Effect of leaching parameters on extraction of Sc: (**a**) effect of pH; (**b**) effect of temperature; (**c**) effect of MgSO_4_ concentration.

To further evaluate the effect of leaching parameters on the extraction of Sc, experiments were conducted at different pH values, MgSO_4_ concentrations, temperatures, and times (Figure 5). Sc was selected as it represented more than 90% of the value of REEs in the RM [36].

Figure 5a shows the effect of pH and time on the leaching of Sc. The extraction of Sc increased with decreasing pH, and a maximum extraction was reached at a pH of 2. The extraction of Sc increased by about 20% with a decrease in pH from 5 to 2 after 60 min of leaching. The leaching of Sc is highly affected by the free H^+^ ions in solution [37] which are connected with the chemical association between Sc and major elements, especially Fe, Ti, and Al. As was stated in the Introduction, Borra et al. [10] showed that up to 50% of Sc could be extracted from industrial RM without a high dissolution of Fe. To increase further extraction of Sc, a strong acid should be used or preliminary activation with the release of REEs from the solid matrix of iron minerals. The RM-ESPD was already thermally and alkali activated. Therefore, Sc extraction was higher than 50% even at high pH values.

High extraction efficiency at pH values of 3–5 can also be attributed to the presence of Mg^2+^ in the solution. At these pH values, the concentration of free H^+^ ions in the solution is very low and not sufficient for a high extraction rate. Very low Sc extraction from alkali-activated RM was observed at a pH if 3.5 in our previous research [14], there was no Mg addition. Then, Mg^2+^ begins to act as a leaching reagent because it exchanges REEs adsorbed on the RM surface. This enhances the rate of the diffusion stage and, subsequently, whole process rates at pH values higher than 3. However, the effect of time on Sc extraction was much higher. With an increase in time from 5 to 60 min, the Sc extraction increased from 26.5 to 75.6% at a pH of 2 and T = 60 °C. The same can be seen in Figure 5b, where the effect of time and temperature on Sc extraction are shown.

With an increase in temperature from 40 °C to 80 °C, Sc extraction increased from 47.5% to 72.5%, after 60 min of leaching at a pH of 3 (Figure 5b). At the same time, the extraction of Sc increased by about 54% with an increase in time from 5 to 60 min at 80 °C. The lower effect of temperature can be attributed to diffusion as a limiting stage of the process. To enhance the acid leaching process limited by diffusion, the concentration of the free reagent in the solution should be increased. An increase in H^+^ concentration obviously increases the extraction rate, as can be seen in Figure 5a. However, an increase in MgSO_4_ concentration (Figure 5c), does not lead to a significant Sc extraction increase. With an increase in MgSO_4_ concentration from 12 g L^−1^ to 36 g L^−1^, Sc extraction increased from 59.8% to 67.4% after 60 min of leaching, at a pH of 3 and temperature of 60 °C, and almost no difference between results at 24 g L^−1^ and 36 g L^−1^ MgSO_4_ concentrations was seen. It implies that not external but intraparticle diffusion can be the limiting stage of the process. To evaluate this, a kinetics study was conducted.

### 3.3. Kinetics Study of Sc Leaching with MgSO_4_

The shrinking core model (SCM) is commonly used to describe the heterogeneous reaction between liquid media reagent and a porous solid compound with a uniform particles size [38,39]. There are three main equations derived for the processes limited by: diffusion through the liquid film (external diffusion, Equation (2)), diffusion through the inert porous solid product of reaction (internal diffusion, Equation (3)), and surface chemical reaction (Equation (4)).

X = k_1_t, (2)

1 − 2/3X − (1 − X)^2/3^ = k_2_t, (3)

1 − (1 − X)^1/3^ = k_3_t, (4)

where k_i_ is the apparent rate constant, X is the degree of Sc extraction, and t is the leaching time.

During extraction of REEs from RM by highly diluted acid, the unreacted core of the raw material (RM particles with the adsorbed REEs, i.e., iron minerals that were shown as the main REEs containing phase in red muds) shrinks to the center leaving behind the inert solid product, i.e., iron minerals without adsorbed REEs. The deeper the reagent goes, the thicker the porous layer of iron minerals around the core that still contains REEs. Eventually, if the iron minerals cannot be dissolved, as happens at pH > 3, then the extraction of REEs from the nonporous core (solid matrix) fails to complete. The leaching rate of REEs can also be limited by external diffusion when all the reagent that is transferred to the particle surface immediately reacts. It relates to the fact that the H^+^ ion concentration is relatively low when highly diluted acids are used. The surface chemical reaction very rarely limits the leaching of REEs. However, high temperature significantly accelerates the leaching rate of iron minerals [14], which leads to a higher extraction efficiency of REEs from the solid matrix.

To evaluate the limiting stage of Sc extraction from the RM-ESPD by MgSO_4_, the fitting of experimental data in Figure 5 to the Equations (2)–(4) was conducted. The fitting accuracy was determined by estimating the coefficient of determination (R^2^), as shown in Table 5 (equations with the higher R^2^ are highlighted in bold).

As can be seen from Table 5, the surface reaction SCM equation (Equation (4)) is poorly suited to describe this leaching process, since the coefficient of determination is the lowest at every T, pH, and C_MgSO4_. It is also obvious that the kinetic data, best of all, correspond to the internal diffusion shrinking core model. However, at low temperatures (40–50 °C), low C_MgSO4_ (12 g L^−1^), and at a high pH, kinetic data are better described by the external diffusion SCM equation (Equation (2)). This indicates that the higher the extraction rate, the thicker the porous layer of iron minerals around the unreacted core. When the solid product layer reaches a certain value, the intraparticle diffusion rate becomes slower than the external one.

**Figure 6:**
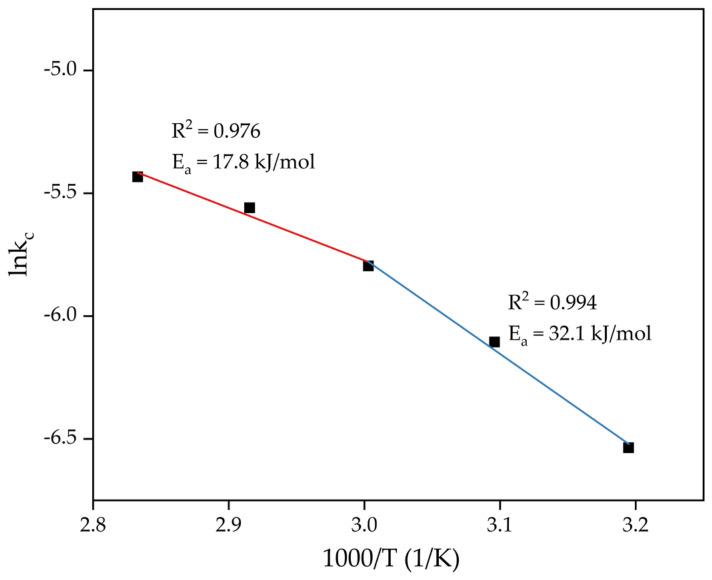
The dependence of lnk_c_ on inverse temperature (1000/T).

The apparent rate constant k_c_ for each temperature obtained by fitting the experimental data of leaching RM-ESPD to the SCM Equation (Equation (3)) (Figure 5b) was used for E_a_ determination. Arrhenius plots for the dependence of lnk_c_ on inverse temperature (Figure 6) were constructed. Building a straight-line y = ax + b in this plane of coordinates allowed us to find the coefficient a, which determines the slope of the straight line. Using the Arrhenius equation (Equation (5)) and knowing the slopes of the straight lines, the apparent activation energy can be obtained as:

k_c_ = Ae^−Ea/RT^, (5)

where A is the Arrhenius constant, min^−1^; E_a_ is the apparent energy of activation, kJ mol^−1^; R is the universal gas constant, J mol^−1^ K^−1^; and T is the temperature, K.

Figure 6 shows that there is a distinct change in the mechanism of leaching at 60 °C (1000/T ≈ 3). At higher temperatures, the E_a_ value was lower. The very low E_a_ values are common for a leaching process limited by intraparticle diffusion [38]. It confirms the SCM data (Table 5), where at high temperatures, Equation (3) was more suitable to describe the leaching process. Moreover, according to the literature data [15], a low value of activation energy has commonly been obtained for REE leaching. In addition, external diffusion has been reported to be the rate-limiting stage of REE extraction from an ion-adsorption type rare-earth ore by MgSO_4_ at a pH of 5.7 [26]. It can be concluded that, at lower pH values and higher temperatures of RM-ESPD leaching by acid in the presence of MgSO_4_, the external diffusion is faster than diffusion through the product layer, because the faster leaching rate leads to the formation of a thicker product layer.

### 3.4. Solid Residue Characterization

**Figure 7:**
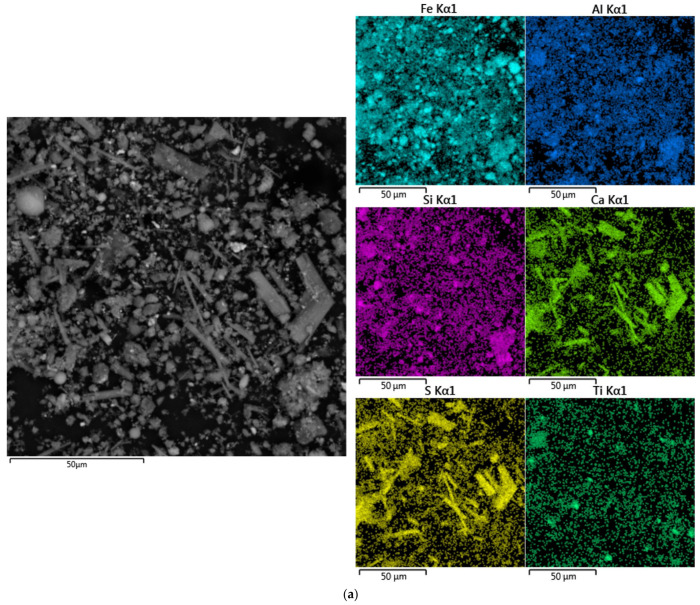

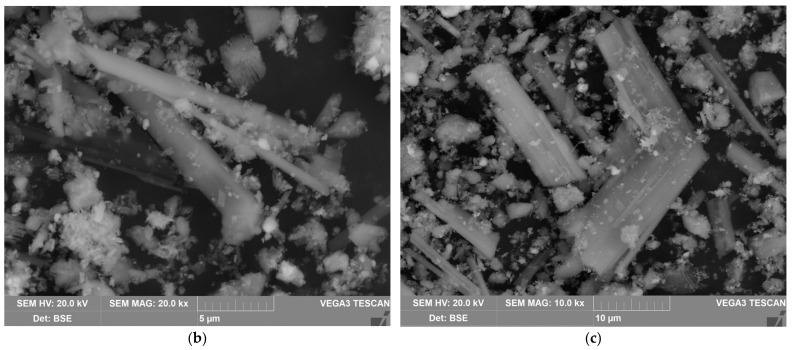
BSE images of: (**a**) The solid residue surface and elemental distribution of major elements (EDS mapping); (**b**) particles of the solid residue at a magnitude of 20,000×; (**c**) particles of the solid residue at a magnitude of 10,000×.

The XRD analysis of the solid residue (Figure 1) shows that RM-ESPD acid leaching with MgSO_4_ leads to the formation of gypsum as the new mineral phase. Gypsum particles can also be seen in Figure 7, where the SEM-EDX analysis of the solid residue is shown. These gypsum particles have a needle shape morphology (Figure 7b,c), and they are bigger than the raw red muds particles. Therefore, some increase in the particle size distribution can be seen in Figure 3. The mechanism of gypsum formation is as follows: The calcium containing katoite and cancrinite are dissolved by acid, then, Ca^2+^ reacts with SO_4_^2−^ ions in the solution with the formation of insoluble gypsum. A high amount of gypsum in solid residue obtained after RM leaching by H_2_SO_4_ has been demonstrated previously in the literature [15]. CaSO_4_ can further hinder the dissolution of REEs, as it encloses (Figure 7a) partially or totally the particles of the raw RM. The co-precipitation of REEs with CaSO_4_ can also occur according to earlier studies [40] because they are physically entrapped. The dissolution of katoite and cancrinite also lead to an increase in pH that results in the REE hydrolysis. Therefore, the use of chloride or nitrate medium and preliminary Ca leaching should increase the REE extraction. However, the high amount of Ca and Al in the solution complicates the subsequent enrichment process of REEs [41].

## 4. Conclusions

In this article, we studied the possibility of selective REE leaching from RM obtained by the water-alkali leaching of the sintering process electrostatic precipitator dust using MgSO_4_ as a leaching reagent. The concentration of REEs in such RM is about two times higher than in the industrial Bayer RM, i.e., 240 mg kg^−1^ Sc vs. 90–120 mg kg^−1^ Sc. Moreover, this RM was alkali and thermally activated, and its particles were very fine (80% lower than 10 μm). As a result, high REE extraction efficiency was obtained even under mild leaching conditions. More than 80% of Sc was extracted at pH = 2, T = 80 °C, L/S ratio of 10, C_MgSO4_ of 24 g L^−1^, and leaching time of 60 min. The Fe extraction at these conditions was 7.7%. Increasing the pH to 4 leads to a decrease in Sc extraction to 63.5% and Fe extraction to 0.03%. The dissolution of other major components was also significantly reduced at a pH of 4. Increasing the pH to 6 leads to a very low extraction of REEs (<15%).

The kinetics study using the shrinking core model (SCM) has shown that diffusion through a product layer is a rate-limiting stage of the process at high temperature (>60 °C) and low pH (<3), whereas, at lower temperatures and higher pH values, the leaching rate is limited by diffusion through the liquid film. The formation of needle-shaped gypsum particles was revealed using SEM analysis. CaSO_4_ can further hinder the dissolution of Sc from RM by the formation of insoluble solid products on the surface of the RM and co-precipitation.

## Figures and Tables

**Table 1:** Mineralogical composition of the red mud obtained by water-alkali leaching of electrostatic precipitator dust arising from the bauxite sintering process (RM-ESPD).

Phase	Content, wt.%
Hematite	56.3
Katoite	18.5
Cancrinite	12.6
Chamosite	6.9
Lepidocrocite	5.7

**Table 2:** Chemical composition of the RM-ESPD.

Compounds	Fe_2_O_3_	SiO_2_	CaO	Al_2_O_3_	TiO_2_	Na_2_O	CO_2_	K_2_O	MgO	MnO	SO_3_	P_2_O_5_	LOI ^1^
Content, wt.%	58.30	9.26	9.01	7.79	2.97	3.41	2.27	0.95	0.54	0.24	0.19	0.13	4.62

^1^ LOI was determined by calcination at 1000 °C for 60 min.

**Table 3:** Contents of REEs in the RM-ESPD.

Element	Sc	Y	La	Ce	Pr	Nd	Nb	Sm
Content, mg kg^−1^	240	557	614	1058	183	615	264	109

**Table 4:** Selected elements concentration in the leaching solutions (mg L^−1^) obtained at pH values of 2, 4, and 6; temperature of 80 °C; leaching time of 60 min; L/S ratio of 10:1; and MgSO_4_ concentration of 24 g L^−1^.

Element	pH 2	pH 4	pH 6
**Na**	2706.0	2755.0	2381.3
**K**	517.3	439.4	281.3
**Ca**	2026.7	1668.8	1694.8
**Al**	2401.3	360.5	341.3
**Fe**	4500.7	19.9	14.6
**Si**	3373.3	914.4	286.3
**Ti**	466.7	3.1	2.6
**Mn**	200.0	125.0	51.8
**Li**	33.0	24.0	20.1
**Co**	10.0	4.4	1.3
**Ni**	26.7	13.8	9.7
**Cu**	26.0	9.4	3.2
**Zn**	120.0	112.5	88.1
**Cd**	86.7	81.3	15.5
**Pb**	100.0	50.0	0.5
**Ga**	9.8	0.7	0.2
**Sc**	19.5	15.2	0.6
Y	24.4	17.2	2.0
**Nb**	1.7	0.2	0.1
**La**	44.0	37.1	8.3
**Ce**	66.7	50.0	5.5
**Pr**	12.2	6.9	1.1
**Nd**	42.7	25.2	3.4
**Sm**	7.3	4.6	0.4
**Th**	6.1	0.1	0.1
**U**	0.8	0.4	0.2

**Table 5:** Shrinking core model (SCM) equations fitting results.

SCM Equation	R^2^											
	pH				Temperature, °C					C_MgSO4_, g L^−1^		
	2	3	4	5	40	50	60	70	80	12	24	36
Equation (2)	0.930	0.962	**0.978**	**0.991**	**0.972**	**0.970**	0.962	0.954	0.940	**0.981**	0.962	0.954
Equation (3)	**0.988**	**0.965**	0.912	0.889	0.935	0.942	**0.965**	**0.965**	**0.985**	0.876	**0.965**	**0.964**
Equation (4)	0.915	0.954	0.977	0.988	0.957	0.963	0.954	0.946	0.920	0.980	0.954	0.945

## Data Availability

Data sharing not applicable.
